# Normal mitochondrial respiratory function is essential for spatial remote memory in mice

**DOI:** 10.1186/1756-6606-1-21

**Published:** 2008-12-16

**Authors:** Daisuke Tanaka, Kazuto Nakada, Keizo Takao, Emi Ogasawara, Atsuko Kasahara, Akitsugu Sato, Hiromichi Yonekawa, Tsuyoshi Miyakawa, Jun-Ichi Hayashi

**Affiliations:** 1 Graduate School of Life and Environmental Sciences, University of Tsukuba, Tsukuba, Ibaraki 305-8572, Japan; 2 Division of Systems Medical Science, Institute for Comprehensive Medical Science, Fujita Health University, 1-98 Dengakugakubo, Kutsukake-cho, Toyoake, Aichi 470-1192, Japan; 3 Genetic Engineering and Functional Genomics Group, Frontier Technology Center, Graduate School of Medicine, Kyoto University, Yoshida-Konoe-cho, Sakyo-ku, Kyoto 606-8501, Japan; 4 Department of Cell Physiology and Metabolism, University of Geneva Medical School, Rue Michel Servet 1, 1211 Geneva 4-CH, Switzerland; 5 Department of Laboratory Animal Science, The Tokyo Metropolitan Institute of Medical Science, 3-18-22 Honkomagome, Bunkyo-ku, Tokyo 113-8613, Japan

## Abstract

**Background:**

Mitochondrial DNA (mtDNA) with pathogenic mutations has been found in patients with cognitive disorders. However, little is known about whether pathogenic mtDNA mutations and the resultant mitochondrial respiration deficiencies contribute to the expression of cognitive alterations, such as impairments of learning and memory. To address this point, we used two groups of *trans*-mitochondrial mice (mito-mice) with heteroplasmy for wild-type and pathogenically deleted (Δ) mtDNA; the "low" group carried 50% or less ΔmtDNA, and the "high" group carried more than 50% ΔmtDNA.

**Results:**

Both groups had normal phenotypes for not only spatial learning, but also memory at short retention delays, indicating that ΔmtDNA load did not affect learning and temporal memory. The high group, however, showed severe impairment of memory at long retention delays. In the visual cortex and dentate gyrus of these mice, we observed mitochondrial respiration deficiencies, and reduced Ca^2+^/calmodulin-dependent kinase II-α (α-CaMKII), a protein important for the establishment of spatial remote memory.

**Conclusion:**

Our results indicated that normal mitochondrial respiratory function is necessary for retention and consolidation of memory trace; deficiencies in this function due to high loads of pathogenically mutated mtDNA are responsible for the preferential impairment of spatial remote memory.

## Background

Mitochondria are intracellular organelles containing their own genomes (mtDNA), and playing a crucial role in ATP production through oxidative phosphorylation. Mammalian mitochondria have multiple copies of mtDNA (10^3 ^~ 10^4 ^copies/cell) that is replicated and expressed within the organellar system [1,2]. Mammalian mtDNA encodes 13 polypeptides, which are essential subunits of complexes I, III, IV, and V for oxidative phosphorylation on the inner mitochondrial membrane, and 22 tRNAs and 2 rRNAs, which are necessary for the translation of these 13 polypeptides. The remaining mitochondrial proteins for oxidative phosphorylation, metabolic enzymes, DNA and RNA polymerases, and ribosomal proteins are all encoded by the nuclear genome [1].

The accumulation of pathogenic mtDNAs with large-scale deletion or point mutations, and the resultant mitochondrial respiration deficiencies are associated with a wide variety of disorders, such as mitochondrial diseases, neurodegenerative diseases, diabetes, and aging [1]. Moreover, dementia and ataxia are found in patients with traditional mitochondrial diseases caused by accumulation of mutated mtDNAs, such as MELAS (mitochondrial encephalopathy, lactic acidosis, and stroke-like episodes), MERRF (myoclonic epilepsy and ragged red fibers), KSS (Kearns-Sayre Syndrome), CPEO (chronic progressive external ophthalmoplegia), and NARP (neurogenic muscle weakness, ataxia, and retinitis pigmentosa) [3-8], and the same mutated mtDNAs have been identified in patients with dementia, ataxia, and Alzheimer's disease [9,10]. Besides, there appears to be a relationship between mtDNA polymorphisms and cognitive function in humans [11]. These findings suggest that mtDNAs with both pathogenic mutations and polymorphisms contribute to various cognitive disorders, thus leading to dementia, and ataxia. It has been demonstrated that polymorphisms, at least, in mtDNAs are responsible for changes in mammalian cognitive function, since the exchange of mtDNAs between NZB/BINJ and CBA/H mice affected their learning and exploration processes [12]. However, there is no direct experimental evidence that mitochondrial dysfunction induced by pathogenic mtDNAs results in cognitive disorders, because no procedures are available for the direct introduction of mutagenized mammalian whole mtDNA into the mitochondria of living cells, or even into isolated mitochondria.

*Trans*-mitochondrial mice carrying pathogenic mtDNAs are very useful for addressing whether mitochondrial respiration deficiencies induced by the mtDNA mutations are responsible for cognitive alterations, and, if so, how they affect brain function. Mito-mice are a type of *trans*-mitochondrial mice generated by the direct introduction of mitochondria carrying ΔmtDNA isolated from cultured cells into normal pronuclear embryos by a cytoplast fusion technique [13]. The ΔmtDNA has an expanded deletion of 4696 bp, from nucleotide position 7,759 in the *tRNA*^*Lys *^gene to position 12,454 in the *ND5 *gene [13], and is similar to the "common deletion" found in KSS, and CPEO patients [14]. The great advantages of mito-mice are that they all share exactly the same nuclear genomic background (C57BL6/J; B6), and their genetic variation is restricted to the proportions of introduced pathogenic ΔmtDNA. Therefore, mito-mice could provide direct evidence that mitochondrial respiration deficiencies induced by mtDNA accumulation are sufficient in themselves for expression of the clinical phenotypes observed in patients with mutated mtDNA.

We used these mito-mice to examine the direct relationship between mutated mtDNA and cognitive alteration, and we succeeded in showing that mutated mtDNA and the resultant mitochondrial respiration deficiencies were responsible for impairments of spatial remote memory. Furthermore, our study demonstrated that mitochondrial respiration deficiencies gave rise to downregulation of α-CaMKII, which is important for the establishment of spatial remote memory in the hippocampus and cortex. These findings suggested that mito-mice with impaired spatial remote memory would be valuable models for understanding the molecular mechanisms of cognitive alteration of mitochondrial origin.

## Results

### Behavioral and physical features of mito-mice

**Figure 1:**
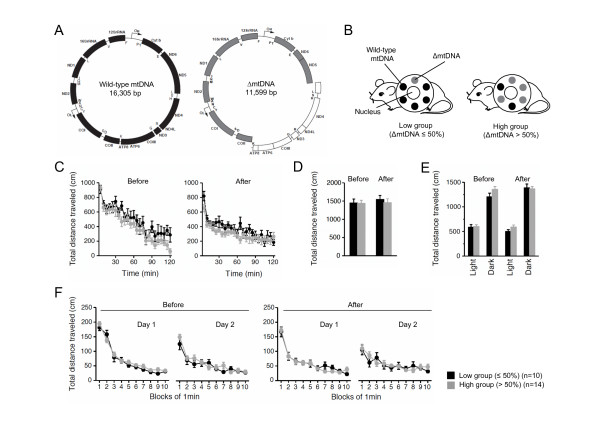
**Genetic characterization and locomotor activity of mito-mice before and after cognitive analyses**. (A) Gene map of mouse wild-type mtDNA (black) and ΔmtDNA (gray). The white arc in the ΔmtDNA indicates the deleted region expanded from the tRNA^*Lys *^to *ND5 *genes. (B) Two groups of mito-mice were used in the experiment. Mito-mouse population (n = 24) was divided into two groups, low (n = 10), carrying 50% or less ΔmtDNA in their tails (at age 4 weeks), and high (n = 14), carrying more than 50%. The two groups shared the same nuclear genome background (C57BL/6). (C-F) To examine locomotor activity in the low group (black), and high (gray) group, four kinds of traditional behavioral test, the open field test (C) (*P *= 0.1638 in before, *P *= 0.1502 in after), elevated plus maze test (D) (*P *= 0.9589 in before, *P *= 0.5703 in after), light-dark transition test (E) (before: *P *= 0.8434 in light side, *P *= 0.1114 in dark side; after: *P *= 0.1379 in light side, *P *= 0.8239 in dark side), and Porsolt forced-swim test (F) (before: *P *= 0.3685 in day 1, *P *= 0.2449 in day 2; after: *P *= 0.7663 in day 1, *P *= 0.7735 in day 2), were performed before and after cognitive analyses. Results of total distance analyses indicated that there were no differences in locomotor activity between the low and high groups. All values are means ± SE.

Mito-mice that are models for mitochondrial diseases carry both wild-type mtDNA and ΔmtDNA (Figure 1A), and the proportions of ΔmtDNA differ among individuals [13]. Not all mito-mice are useful for examining cognitive alteration, because in mice with severe mitochondrial disease phenotypes due to high loads of ΔmtDNA (approximately more than 80%), the abnormal cognitive function can be overshadowed by other abnormalities in the phenotype. In fact, a high load of ΔmtDNA induces locomotor defects in male mito-mice, leading to impaired mating activity [15]. Furthermore, the proportion of ΔmtDNA in various tissues of mito-mice increases progressively with aging, and the mice begin to show the onset of mitochondria-driven diseases other than those manifested cognitively [16]; we therefore had to finish all of our behavioral analyses before ΔmtDNA accumulated to more than approximately 80%.

To minimize these problems, we selected mito-mice carrying 21% to 76% ΔmtDNA (n = 30), as determined by analysis of the mtDNA in their tails at age 4 weeks. The proportions of ΔmtDNA in other tissues could be deduced from those in the tail samples without the need to sacrifice the mito-mice, because the proportions are very similar throughout all the tissues of individual mito-mice at age 4 weeks [13,17]. We then classified the mito-mice into two groups: low (n = 13), carrying 50% or less ΔmtDNA in their tails, and high (n = 17), carrying more than 50% (Figure 1B). From our previous studies, we expected that the mito-mice in the low group would maintain normal mitochondrial respiration activity, and would thus be quite healthy during the behavioral analyses, whereas those in the high group would show slight mitochondrial respiration abnormalities, and resultant lactic acidosis, one of the markers for the onset of mitochondrial diseases, at about the time the study ended [13,15]. Since the genetic variation in the mito-mouse population was limited to the proportions of exogenously introduced ΔmtDNA, the low group was used as experimental normal controls.

**Figure 2:**
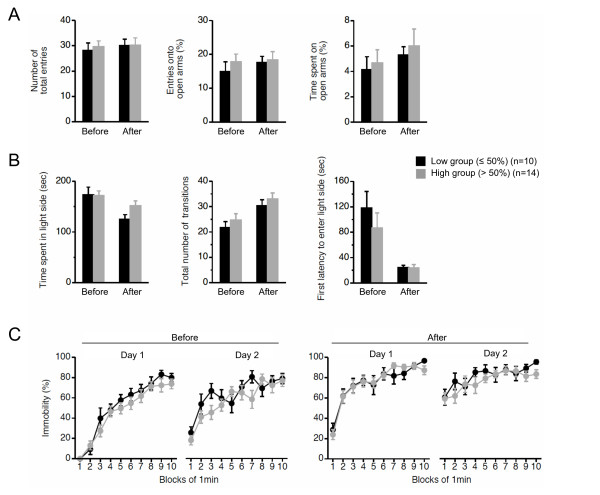
**Behavioral analyses of mito-mice before and after cognitive analyses**. To examine behavioral performances in the low group (black), and high (gray) group, three kinds of traditional behavioral tests were performed before and after cognitive analyses. (A) Elevated plus maze test. There was no difference in phenotypes of anxiety-related behavior between the low and high groups at the two time points (number of total entries: *P *= 0.6823 in before, *P *= 0.9775 in after; entries onto open arms: *P *= 0.4360 in before, *P *= 0.8226 in after; time spent on open arms: *P *= 0.7157 in before, *P *= 0.6756 in after). (B) Light-dark transition test. There was no difference in phenotypes of anxiety-related behavior between the low and high groups at the two time points (time spent in light side: *P *= 0.9125 in before, *P *= 0.0625 in after; total number of transitions: *P *= 0.4083 in before, *P *= 0.4569 in after; first latency to enter light side: *P *= 0.3812 in before, *P *= 0.9798 in after). (C) Porsolt forced-swim test. There was no difference in phenotypes of depressant-related behaviors between the low and high groups at the two time points (before: *P *= 0.1479 in day 1, *P *= 0.0526 in day 2; after: *P *= 0.9900 in day 1, *P *= 0.3132 in day 2). All values are means ± SE.

Before we focused on cognitive function in the mito-mice, we examined the locomotor activity and physical performance of the two groups (n = 10 as a low group, and n = 14 as a high group), because the results of spatial learning and memory tests for the study of cognitive function are easily affected by locomotor and physical abnormalities. We therefore performed an open field test (Figure 1C), elevated plus maze test (Figure 1D and 2A), light-dark transition test (Figure 1E and 2B), and Porsolt forced-swim test (Figure 1F and 2C), before and after the cognitive analyses. There were no significant differences between the low and high groups in terms of locomotor activity and physical performance, including anxiety-, and depression-related behaviors, at the two time points (Figure 1C–1F and 2). From these results, we concluded that the two groups of mito-mice could be used as an *in vivo *model to confirm the direct relationship between mutated mtDNA and cognitive alteration.

### Mito-mice showed abnormalities at long retention delays in cognitive analyses

**Figure 3:**
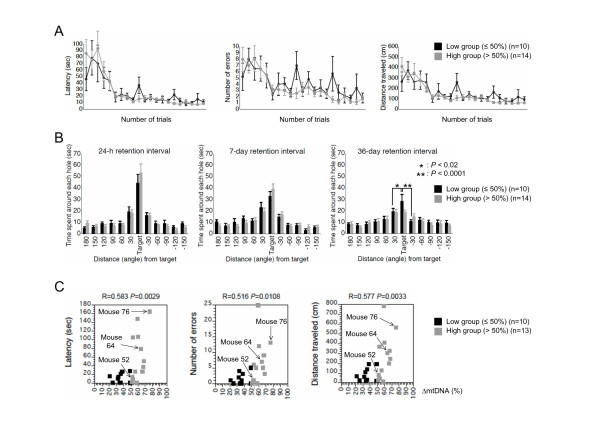
**Spatial learning and memory analyses using Barnes circular maze test in mito-mice**. (A) Training course. Latency (*P* = 0.7381), number of errors (*P* = 0.5051), and distance traveled (*P* = 0.7885) to target hole for low (black) and high (gray) groups were plotted. (B) Probe tests at 24-h, 7-day, and 36-day after the last training. There was no significant difference in the probe test at 24-h (between the target hole and right side hole, *P* < 0.005 in the low and *P* < 0.001 in the high groups; between the target hole and left side hole, *P* < 0.05 in the low and *P* < 0.002 in the high groups) and 7-day retention intervals (between the target and right side holes, *P* < 0.001 in the low and *P* < 0.001 in the high groups; between the target and left side holes, *P* < 0.05 in the low and *P* < 0.002 in the high groups). In the probe test at the 36-day retention interval, single and double asterisks indicate significant differences between the target and right side holes in the low group (*P* < 0.02 in the low and *P* = 0.9804 in the high groups) and between the target and left side holes in the low group (*P* < 0.0001 in the low and *P* = 0.2106 in the high groups), respectively. (C) A single retraining after the probe test at the 36-day retention interval. Each score of individual mito-mice in the low (black) and high (gray) groups were plotted against the proportion of ΔmtDNA in the tails at age 4 weeks. Mice 52, 64, and 76 carried 52%, 64%, and 76% ΔmtDNA, respectively. Pearson’s product-moment correlation coefficients and the associated probabilities are indicated as R and *P*, respectively. All values are means ± SE.

To examine cognitive function in mito-mice (n = 10 as a low group, and n = 14 as a high group), we performed Barnes circular maze tests (see Methods), which can reveal spatial learning and memory ability without the effects by difference in motor coordination or emotional aspects. During the course of training (21 trials for 7 days), two groups of mito-mice learned to locate a single target among 12 holes. Underneath the target hole was an escape box containing shredded paper. We measured three parameters, latency, number of errors, and distance traveled until they located the target hole. In both groups, all parameters were progressively reduced with training, indicating acquisition of spatial learning (Figure 3A). Through the training trials, there were no statistical differences between the two groups in latency, number of errors, and distance traveled to the target hole (Figure 3A). These results indicated that a high load of ΔmtDNA was not required for acquisition of spatial memory, although we could not rule out the possibility that a load of more than 80% – the pathogenic threshold for expression of systemic mitochondrial disease phenotypes – would induce impairment of spatial learning.

To assess the spatial memory of mito-mice at long retention delays, we performed probe tests at 24-h, 7-day, and 36-day retention intervals after the last training trial (see Methods). Since the probe tests were conducted without an escape box, there was no box for them to escape into under the target hole. If the mouse had memorized a target, it would visit the hole where the escape box had previously been located and spend a long time around it. In the probe tests at the 24-h and 7-day retention intervals, which we used to examine temporal memory ability, there were no statistical differences in the times spent around the target hole by the two groups (Figure 3B, left and center panels). However, in the probe test at the 36-day retention interval, which was used to examine remote memory ability, the high group spent significantly less time around the target hole than did the low group (Figure 3B, right panel). These results indicated that abnormalities in spatial remote memory were induced by a high load of ΔmtDNA.

Phenotypic expression of mitochondrial diseases is regulated by a threshold effect of the mutated mtDNAs: that is, most phenotypes of mitochondrial disease appear only when mutated mtDNAs have accumulated to a certain level [17]. If the ΔmtDNA load were responsible for abnormalities in spatial remote memory, then only mito-mice with a high load of ΔmtDNA – beyond a certain threshold – would be likely not to memorize the target hole. To confirm this point, we performed a single retraining of each mito-mouse after the probe test at the 36-day retention interval, and we measured three parameters during the training session: latency, number of errors, and distance traveled to the target hole with the escape box under it (see Methods). Increases in the three parameters were observed in each mito-mouse when the ΔmtDNA load was more than approximately 60% (Figure 3C). The results showed that a high load of ΔmtDNA was definitely responsible for the impairment of spatial remote memory; they also suggested that the pathological threshold for expressing the memory impairment phenotype was approximately 60%.

### Accumulation of ΔmtDNA and resultant mitochondrial respiration deficiencies in brain tissues of mito-mice

**Figure 4:**
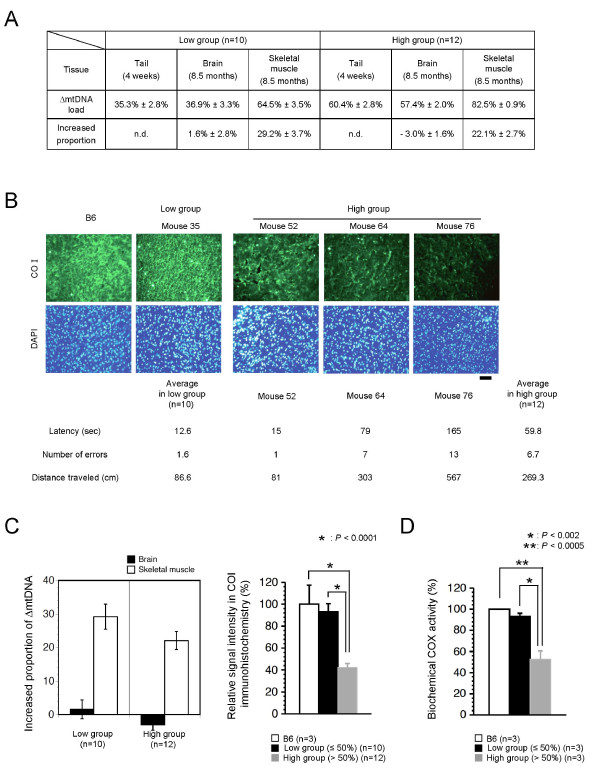
**ΔmtDNA load and mitochondrial respiration in brain tissues**. (A) Accumulation of ΔmtDNA. Because ΔmtDNA is distributed nearly uniformly throughout all the tissues of a mito-mouse at age 4 weeks, we estimated the increased proportion of ΔmtDNA in brain and skeletal muscle tissues after the experiment (at age 8.5 months), based on ∆mtDNA proportions in tail samples at age 4 weeks. (B) Relationship between mitochondrial respiratory function and phenotypic expression of impaired spatial remote memory. Sections of visual cortex from B6, low group, and high group (Mice 52, 64, and 76) were stained with anti-COI antibody and DAPI. Mice 52, 64, and 76 carried 52%, 64%, and 76% ΔmtDNA, respectively, in their tails at age 4 weeks. Numbers under the sections for Mice 52, 64, and 76 indicate scores in the spatial remote memory experiment shown in Figure 3C. (C) Graphic representation of Figures 4A and 4B. In the left graph, bars indicate values obtained from deduction of ΔmtDNA proportion in the tail at age 4 weeks from that in brain (black bar) or skeletal muscle (white bar) tissues at age 8.5 months. In the right graph, quantification of COI positive signals in visual cortex of B6 (white bar), low group (black bar), and high group (gray bar) mice were shown. (*P*=0.5942 in B6 vs low group, *P* < 0.0001 in B6 vs high group, and *P* < 0.0001 in low vs high groups). (D) Biochemical analysis of COX activity in brain tissues. Whole brain samples from B6 (white bar), low group (black bar), and high group (gray bar) were used for the analysis (*P*=0.3469 in B6 vs low group, *P* < 0.0005 in B6 vs high group, and *P* < 0.002 low vs high groups). All values are means ± SE. All asterisks indicate significant differences. n.d., not determined.

It was not clear whether the impaired spatial remote memory in the high group was induced by mitochondrial respiration deficiencies due to high load of ΔmtDNA in the brain tissues. To address this point, we first estimated the proportion of ΔmtDNA in brain tissues from the two groups. We previously reported that ΔmtDNA can accumulate over time in cultured cells and various tissues because it has replication advantages [13,16]. In consideration of this feature of ΔmtDNA, we selected a high group of mito-mice in which ΔmtDNA would accumulate only to less than 80%, even when all the analyses in the study were finished. The proportion of ΔmtDNA in the skeletal muscle tissues at age 8.5 months was higher in both groups than in the tail samples at age 4 weeks. The increase in the proportion of ΔmtDNA in the skeletal muscle was 29.2% ± 3.7% in the low group and 22.1% ± 2.7% in the high group (Figure 4A and 4C). The proportion of ΔmtDNA in the brain tissues, however, increased very little or decreased; at age 8.5 months the proportion of ΔmtDNA in the brain ranged from 25% to 53% in the low group (increase compared with tail samples at 4 weeks, 1.6% ± 2.8%) and from 49% to 71% in the high group (increase compared with tail samples at 4 weeks, -3.0% ± 1.6%) (Figure 4A and 4C).

It is well known that the enzymatic activity of mitochondrial respiratory complexes that contain mtDNA-coded subunits is reduced by pathogenic mutations in mtDNA. Reduction in cytochrome *c *oxidase (Complex IV: COX) activity is a pathological marker for mitochondrial diseases of mtDNA derivation, because COX contains three mtDNA-coded subunits, COI, COII, and COIII (see Figure 1A), and the loss of these subunits by mutated mtDNAs can lead to mitochondrial respiration deficiencies and disease phenotypes [1]. Since the ΔmtDNA loses 6 tRNA genes (see Figure 1A), the pathogenicity of the ΔmtDNA is expressed as lack of all 13 structural gene products which mtDNA codes, resulting in decreased activity of complexes I, III, IV, and V. We therefore examined the immunohistochemical expression of COI protein, one of the COX subunits, in order to visualize mitochondrial respiratory function in brain tissues from the two groups. The COI expression was normal in visual cortical sections from the low group, because we observed the same level of COI expression as in normal B6 mice (Figure 4B and 4C). In contrast, visual cortical sections from the high group showed reduced COI expression; differences in COI expression level – i.e., very slight, mild, and moderate stain-loss – were seen in Mice 52, 64, and 76, which carried 55%, 63%, and 71% ΔmtDNA, respectively, in their brain tissues (Figure 4B and 4C). Then, we performed biochemical analysis for COX activity in brain tissues from the two groups. There was no significant difference in the COX activity between normal B6 mice and low group mito-mice (Figure 4D). However, we observed a half of the COX activity in whole brain tissues from the high group (Figure 4D). From these results, together with the results of retraining after the last probe test (Figure 3C), we confirmed that the mitochondrial respiration deficiencies due to high load of ΔmtDNA were consistent with the phenotypic expression of impairments of spatial remote memory. The three parameters – latency, number of errors, and distance traveled to the target hole – increased gradually in the order of Mice 52, 64, and 76 (Figure 4B), reflecting the hypothesis that phenotypic expression and severity of impairment of remote memory depended upon the degree of mitochondrial respiration deficiency.

### Expression of α-CaMKII protein in mito-mice

**Figure 5:**
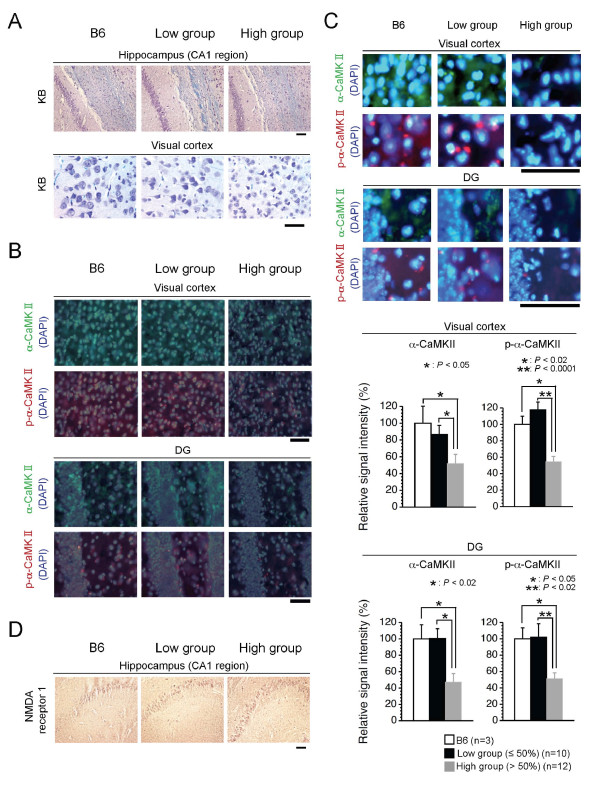
**Histological and immunohistochemical analyses of brain tissues**. (A) Histological observations in the hippocampus (CA1 region) and visual cortex. Paraffin sections of brain tissues were stained with Kluver-Barrera (KB) stain. (B) Immunohistochemical observations in visual cortex and DG. Frozen sections of brain tissues were stained with anti-α-CaMKII (green) or anti-p-α-CaMKII (red) antibody, and with DAPI (blue). a-CaMKII and DAPI signals, or p-α-CaMKII and DAPI signals, were respectively merged. (C) Amplified images and quantification of α-CaMKII and p-α-CaMKII in visual cortex and DG areas. A part of each image in Figure 5B was representation as amplified images. In the graphic representation, quantification of α-CaMKII and p-α-CaMKII positive signals in visual cortex and DG of B6 (white bar), low group (black bar), and high group (gray bar) were shown (a-CaMKII in visual cortex: *P* = 0.5333 in B6 vs low group, *P* < 0.05 in B6 vs high group, and *P* < 0.05 in low vs high groups; p-a-CaMKII in visual cortex: *P* = 0.2884 in B6 vs low group, *P* < 0.02 in B6 vs high group, and *P* < 0.0001 in low vs high groups; a-CaMKII of DG: *P* = 0.9775 in B6 vs low group, *P* < 0.02 in B6 vs high group, and *P* < 0.02 in low vs high groups; p-a-CaMKII of DG: *P* = 0.9263 in B6 vs low group, *P* < 0.05 in B6 vs high group, and *P* < 0.02 in low vs high groups). Values are the means ± SE. Asterisks indicate significant differences. (D) Immunohistochemical observations in the hippocampus (CA1 region). Paraffin sections of brain tissues from B6, low group, and high group mice were stained with anti-NMDA receptor 1 antibody. All scale bars, 50 µm.

Since it has been suggested that temporal and remote memory is regulated, respectively, as hippocampal and cortex-hippocampal tasks, the results of the Barnes circular maze tests suggested that a high load of ΔmtDNA affected cortex-hippocampal tasks, rather than hippocampal ones. However, the mechanisms by which mitochondrial respiration deficiencies of the brain tissues induced abnormalities selectively in cortex-hippocampal tasks were unclear. We therefore compared histological changes in the hippocampus and visual cortex between the two groups. No marked histological abnormalities in cell number and death were seen in either group, with the exception of the fact that some of the neuronal cells in the visual cortical sections from high group mice were slightly smaller than those from low group mice (Figure 5A). These observations indicated that mitochondrial respiration deficiencies, at least in the high group expressing impairment of spatial remote memory, did not affect cell quantity and formation in the visual cortex and hippocampus, although a very high load of ΔmtDNA (more than 80%) might have induced some histological abnormalities.

**Figure 6:**
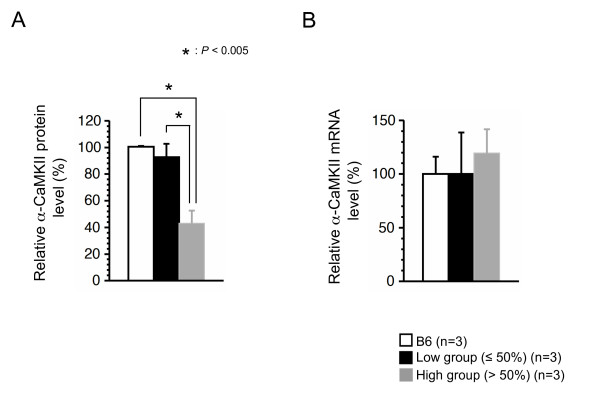
**Quantification of α-CaMKII protein and mRNA in visual cortex**. (A) Quantification of α-CaMKII protein in the visual cortex by Western blotting analysis. Extracted protein of the visual cortex from B6, low group, and high group were blotted with an anti-α-CaMKII antibody. Bars indicate values of α-CaMKII protein expression in the visual cortex from the B6 (white bar), low group (black bar), and high group (gray bar). In the high group, remarkable downregulation of α-CaMKII proteins (*P *= 0.5148 B6 vs low group, *P *< 0.005 B6 vs high group, *P *< 0.005 low group vs high group) was observed. The β-actin was used as a loading control in the Western blotting. Values are the means ± SE. Asterisks indicate significant differences. (B) Quantification of α-CaMKII mRNA in the visual cortex. Total mRNAs were extracted from frozen samples of the visual cortex from B6, low group and high group, and cDNAs were obtained by a reverse transcription method. Relative expression levels of α-CaMKII mRNA were determined by a real-time monitoring PCR technique. Bars indicate values of relative expression levels of α-CaMKII mRNA in the B6 (white bar), low group (black bar), and high group (gray bar). The β-actin was used as a loading control in the real-time monitoring PCR technique. There was no significant difference among the three groups (*P *= 0.9972 B6 vs low group, *P *= 0.6214 B6 vs high group, *P *= 0.6238 low group vs high group). Values are means ± SE.

α-CaMKII is important for the establishment of remote memory, including for cortical plasticity and consolidation of memory traces in cortical networks [18]. A part of α-CaMKII mRNAs is targeted dendritically, then translated at the site, and then finally matured into constitutively active forms [19]. Since the occurrence of impairment of spatial remote memory was correlated with mitochondrial respiration deficiency due to high load of ΔmtDNA, there was a possibility that mito-mice in the high group possessed some abnormalities in expression and/or dendritic distribution of α-CaMKII protein. To test this, we examined expression of α-CaMKII and phosphorylated-α-CaMKII (p-α-CaMKII) as the translated and constitutively active forms, respectively. In visual cortical sections from the low group, both α-CaMKII and p-α-CaMKII proteins were expressed well and distributed in dendrites and cytoplasm as well as those in the B6 mouse (Figure 5B, 5C and 6A). In contrast, we observed a notable general reduction in both α-CaMKII and p-α-CaMKII protein expression in the visual cortical sections from the high group (Figure 5B, 5C and 6A). However, we did not observed significant difference in the expression levels of α-CaMKII mRNA among the three groups (Figure 6B), although the high group mice possessed a slight increase of the α-CaMKII mRNA level (Figure 6B), probably due to the compensation of decreased α-CaMKII protein (see Figure 6A). These results indicated that mitochondrial respiration deficiency affected translation machinery and/or dendritically-targeting process of α-CaMKII mRNA, rather than phosphorylation of the protein and transcription of the mRNA, thus leading to impairment of spatial remote memory. Moreover, we performed immunohistochemical analyses to visualize α-CaMKII and p-α-CaMKII proteins in section of the dentate gyrus (DG), because the DG is considered to be important in spatial pattern separation [20], and the specific occurrence of adult neurogenesis [21]. Consistent with the visual cortical sections, the levels of expression of both α-CaMKII and p-α-CaMKII proteins in DG sections from the low group were similar to those in the B6 mouse, whereas these expressions in DG sections from the high group were notably reduced (Figure 5B and 5C). These immunohistochemical results suggested that a notable reduction in α-CaMKII and p-α-CaMKII protein production in the visual cortex and DG may have caused the impairment of spatial remote memory. The *N*-methyl-D-aspartate (NMDA) receptor, a major molecule upstream of α-CaMKII, plays an essential role in learning and memory in the hippocampus [22], and degradation of NMDA receptor subunits in the hippocampus contributes to learning and memory defects [23]. We also performed immunohistochemical analyses with anti-NMDA receptor 1, but there was no difference in the expression of NMDA receptor 1 protein between the two groups (Figure 5D).

## Discussion

In healthy mouse models (NZB/BINJ and CBA/H mice) it has been well documented that mtDNA polymorphisms are among the genetic candidates for the control of learning ability [12]. However, there was no direct experimental evidence as to whether mtDNA with pathogenic mutations and the resultant mitochondrial respiration deficiencies are responsible for cognitive alterations. Since mito-mice share the same nuclear genome background, and their genetic variations are restricted to the proportions of pathogenic ΔmtDNA – similar to the case with common deletion types in human diseases [13] – their use could provide unambiguous evidence that pathological phenotypes exclusively observed in mito-mice carrying high loads of ΔmtDNA are caused by ΔmtDNA-induced mitochondrial respiration deficiencies. Our experiments clearly showed that loads of 50% or less ΔmtDNA did not induce mitochondrial respiration deficiencies in the brain tissues, and were also not associated with behavioral abnormalities (Figure 1C–1F, 2, 3, 4B and 4C), whereas loads of more than 50% were able to induce mitochondrial respiration deficiencies, and downregulation of α-CaMKII protein in brain tissues, leading to impairment of spatial remote memory (Figure 3B, 4B, 4C, 4D, 5B, 5C and 6A). We therefore succeeded for the first time in showing experimental evidence that a high load of pathogenically mutated mtDNA and the resultant mitochondrial respiration deficiencies, in the absence of severe mitochondrial disease phenotypes, are responsible for the impairment of spatial remote memory.

It has been considered that remote memory traces are established by the acquired formation of neuronal circuits between the hippocampus and cortex and by the resultant cortical plasticity, whereas learning and temporal memory are regulated by the existing neuronal circuits in the hippocampus [24]. Frankland et al. [18] have been reported the possibility that the α-CaMKII modulates the synaptic events required for the consolidation of memory trace in cortical networks. Heterozygous mice for a null mutation of α-CaMKII showed abnormal cortical plasticity and DG maturation, resulting in impairment of remote memory [18,25]. These mice showed normal hippocampal plasticity, resulting in no impairment of learning, and temporal memory [18,25]. Consistent with these reports, expression levels of α-CaMKII protein were reduced in the cortices and DGs of mito-mice from the high group, and these mice showed impairment of spatial remote memory (Figure 3B, 5B and 5C). In contrast, hippocampal plasticity, which is regulated mainly by existing neuronal circuits in the hippocampus, is required for initial learning, and temporal memory formation [24]. Genetic disruption experiments in mouse models have indicated that NMDA receptors are important not only in the formation of neuronal circuits during brain development, but also in the learning and hippocampus-dependent temporal memory that use these circuits [23,26]. In mito-mice, irrespective of the ΔmtDNA load, the level of expression of NMDA receptor 1 was not altered (Figure 5D); this probably explains why all the mice had normal spatial learning and temporal memory (Figure 3A and 3B). Therefore, the occurrence of mito-mouse phenotypes in which spatial remote memory was impaired but spatial learning and temporal memory were not indicated that mitochondrial respiratory function plays an integral role in the formation of acquired neuronal circuits between the cortex and hippocampus, and/or neuronal plasticity in the cortex, rather than in the use of existent neuronal circuits in the hippocampus.

The pathophysiological mechanisms of mitochondria-related impairment of spatial remote memory can be considered in the following way. When more than 60% ΔmtDNA accumulates in the brain, mitochondrial respiration deficiencies are induced. Reduction and depletion of the mitochondrial energy supply would affect the processes of translation and/or targeting of α-CaMKII mRNA. The reduced α-CaMKII protein would cause impairment of spatial remote memory. There are several possible reasons why α-CaMKII protein was downregulated in the brain tissues of mito-mice with high ΔmtDNA loads. The first is that mitochondrial energy supply is necessary for the dendritically targeted processes of α-CaMKII mRNA. It is well known that the mRNAs of α-CaMKII are targeted to dendrites, and that additional transcription and maturation events occur after this targeting [19]. Many proteins, including cytoskeletal components and ion-motive ATPases, that are critical for the dendritically targeted processes of α-CaMKII mRNA require ATP [27,28]. Thus, one possible reason is that mitochondrial respiration deficiencies could induce abnormalities in the localization of α-CaMKII mRNAs, and the resultant downregulation of its protein form. This possibility was supported by normal level of α-CaMKII mRNA in the high group (Figure 6B). However, the reduction of α-CaMKII protein in high group brain did not always occur specially in dendrites (Figure 5C). The second possibility is that exogenous signals derived from other tissues carrying ΔmtDNA at high levels contribute to the reduction of α-CaMKII protein production in the brain tissues of the mito-mice. Taking this study together with our previous one [16], most tissues in the high group were allowed to accumulate ΔmtDNA with aging, and these tissues fell into mitochondrial respiration deficiency, even though the proportion of ΔmtDNA in the brain tissues did not increase (Figure 4A and 4C). Systemic mitochondrial respiration deficiencies give rise to lactic acidosis, which is one of the diagnostic markers of mitochondrial diseases [1], and the lactate can bind Ca^2+ ^[29]. The onset of lactic acidosis, therefore, could disturb calcium homeostasis and signaling in nerve cells, probably leading to the general reduction of α-CaMKII protein (Figure 5C), although all vital functions that occur via calcium signaling should in fact be affected by the lactic acidosis.

In the brain tissues of the mito-mice, a ΔmtDNA load of more than 60% could induce mitochondrial respiration deficiencies (Figure 4B). The threshold ΔmtDNA load leading to mitochondrial respiration deficiencies in the brain tissues was clearly lower than that in other tissues; a ΔmtDNA load of more than approximately 80% is needed for the occurrence of mitochondrial respiration deficiencies in skeletal and cardiac muscle, and in renal, pancreatic, and testicular tissues [13,15,17,30]. Considering that the brain is a high-energy-demand tissue [1], the lower load of ΔmtDNA was sufficient to induce mitochondrial respiration deficiencies in the brain tissues. In addition, it has been reported that a calcium-regulated signal pathway controls mitochondrial biogenesis, since transgenic mice with overexpression of CaMKIV in their skeletal muscles show enhanced mitochondrial biogenesis, including enhanced mtDNA replication, mitochondrial respiration, and fatty acid metabolism [31]. If mitochondrial biogenesis in the brain is also controlled by a calcium-regulated signal pathway, then a reduction in CaMKII production might participate in mitochondrial respiration deficiencies in brain tissues. The potential involvement of CaMKII in mitochondrial respiration requires further investigation. Moreover, the brain tissues of mito-mice showed little or negative accumulation of ΔmtDNA without neuronal cell death (Figure 4A and 4C) [16], even when the proportions of ΔmtDNA in other tissues increased (Figure 4A and 4C). This phenomenon suggested that the replication frequency of mtDNA molecules differs in each tissue, and that the frequency in the brain is the lowest among various tissues [16]. In human brains, however, it has been reported that deleted mtDNAs accumulate clonally in single cells during aging [32]. Therefore, it remains to be answered why the mouse brain was sensitive to a low load of ΔmtDNA, more than 60%, and why ΔmtDNA did not accumulate in the mouse brain.

The disease phenotypes caused by pathogenic mtDNAs differ among the types of mutation. For instance, mutations in particular tRNA genes are responsible for MELAS, MERRF, and cardiomyopathy, whereas ones in structural genes induce Leigh syndrome, and Leber's disease [33]. We previously generated a novel mouse model (mito-mouse COI) carrying homoplasmic mtDNA with a pathogenic point mutation in the *COI *gene (COImut-mtDNA) [34]. The mito-mice COI looked healthy throughout life, because of the lower pathogenicity of COImut-mtDNA compared with that of ΔmtDNA, but they showed slight mitochondrial respiration deficiencies in all tissues examined [34]. We have obtained preliminary data that mito-mice COI do not have impairment of spatial remote memory, but they have other cognitive function phenotypes (unpublished data). These findings suggest that the occurrence of clinical phenotypes in cognitive function also differs among different types of mutation, and is directly regulated by the intensity of the pathology of mitochondrial respiration. Experimental support for this possibility will require further investigation.

## Conclusion

In summary, our results suggested the importance of mitochondrial respiration for maintaining the plasticity critical for establishment of spatial remote memory. In mitochondrial respiration deficiencies, nerve cells that participate in cortical and hippocampal plasticity are unable to produce α-CaMKII protein; this preferentially leads to impairment of spatial remote memory. The mito-mouse is a very valuable model system for understanding the fundamental roles of mitochondrial respiratory function in retention and consolidation of memory trace, because this mouse is the only mammalian model with impairment of spatial remote memory caused by mitochondrial respiration deficiency. On the basis of our findings, we suggest that some cases of human memory defect are caused by high loads of mutated mtDNA and the resultant mitochondrial respiration deficiencies. We also suggest that improving mitochondrial respiration might be an effective strategy for treating memory disorders, although there are biological differences between such disorders in humans and mice.

## Methods

### Mice

Thirty male mito-mice carrying various proportions of ΔmtDNA were used for this study. The proportion of ΔmtDNA in mito-mice was deduced from tail DNA samples, because it was very similar in all the tissues of the same individual mouse [13,17]. Age-matched male B6 mice (n = 6) were used as normal controls in histological, immunohistochemical, biochemical, Western blotting, and real-time monitoring PCR analyses.

### Estimation of ΔmtDNA proportions by real-time monitoring PCR

Real-time monitoring PCR was used to estimate the proportions of ΔmtDNA in tails mito-mice at age 4 weeks and in brain and skeletal muscle tissues of mito-mice after all behavioral analyses (at age 8.5 months). It was performed with a TaqMan PCR reagent kit and an ABI PRISM 7900HT sequence detection system (Applied Biosystems, Foster City, CA). To estimate the absolute copy number of wild-type mtDNA and ΔmtDNA, we used the standard curve method. The standard curve for the assay was calculated using a series of 10-fold dilution of titrated synthetic standard DNA. Each measurement was repeated three times, and the proportions of ΔmtDNA and total mtDNA were calculated. The primer set specific for ΔmtDNA was TTTCACTATGAAGCTAAGAGCGTTAACCT and GGTGGAATCGGACCAGTAGGA. The reporter dye 6-carboxyfluorescein (FAM)-labeled TaqMan minorgroove-binder probe specific for ΔmtDNA was AACTGGTGTATGGAGATTT. The primer set specific for wild-type mtDNA was AACCTGGCACTGAGTCACCA and GGGTCTGAGTGTATATATCATGAAGAGAAT. The reporter dye FAM and the quencher dye 6-carboxy-tetramethyl-rhodamine (TAMRA)-labeled probe was TCTGTAGCCCTTTTTGTCACATGATC.

### Behavioral analysis

#### Animals and experimental design

Mice were housed four per cage in a room with a 12-hr light-dark cycle (lights on at 7:00 a.m.) with access to food and water ad libitum. Behavioral testing was performed between 9:00 a.m. and 6:30 p.m. After the tests, the apparatus were cleaned with super hypochlorous water to prevent a bias due to olfactory cues. All behavioral tests were conducted in a manner similar to those described previously [35], and performed with male mice between 5 and 8 months after birth. All behavioral testing procedures were approved by the Animal Care and Use Committee of Kyoto University Graduate School of Medicine.

#### Open field test

Open field test was performed with male mice before (at age 5 months) and after (at age 8 months) cognitive analyses, respectively. Each mouse was placed in the center of the open field apparatus (40 × 40 × 30 cm; Accuscan Instruments, Columbus, OH). Total distance traveled (in cm), and number of fecal boli were recorded. Data were collected for 120 min.

#### Elevated plus maze test

Elevated plus maze test was performed with male mice before (at age 5 months) and after (at age 8 months) cognitive analyses, respectively. The elevated plus maze (O'Hara & Co., Tokyo, Japan) consisted of two open arms (25 × 5 cm) and two enclosed arms of the same size, with 15-cm high transparent walls. The arms and central square were made of white plastic plates, and were elevated to a height of 55 cm above the floor. To minimize the likelihood of animals falling from the apparatus, 3-mm high plastic ledges were provided for the open arms. Arms of the same type were arranged at opposite sides to each other. Each mouse was placed in the central square of the maze (5 × 5 cm), facing one of the closed arms. Mouse behavior was recorded during a 10-min test period. The number of entries onto, and the time spent on open and enclosed arms, were recorded. For data analysis, we used the following four measures: total distance traveled (cm), the number of total entries, the percentage of entries onto the open arms, and the time spent on the open arms (s). Data acquisition and analysis were performed automatically using Image EP software (see 'Image analyses').

#### Light-dark transition test

Light-dark transition test was performed with male mice before (at age 5 months) and after (at age 8 months) cognitive analyses, respectively. The apparatus used for the light/dark transition test consisted of a cage (21 × 42 × 25 cm) divided into two sections of equal size by a partition containing a door (O'Hara & Co., Tokyo, Japan). One chamber was brightly illuminated (390 lux), whereas the other chamber was dark (2 lux). Mice were placed into the dark side, and allowed to move freely between the two chambers with the door open for 10 min. Total distance traveled, time spent in each side, total number of transitions between chambers, and first latency to enter the light side were recorded automatically.

#### Porsolt forced-swim test

Porsolt forced-swim test was performed with male mice before (at age 5 months) and after (at age 8 months) cognitive analyses, respectively. The apparatus consisted of four plastic cylinders (20 cm height × 10 cm diameter). The cylinders were filled with water (23°C) up to a height of 7.5 cm. Mice were placed into the cylinders, and their behavior recorded over a 10-min test period for 2 days. Data acquisition and analysis were performed automatically, using Image PS software (see 'Image analyses'). The total distance traveled, and the percentage of immobility were measured by Image OF software (see 'Image analyses') using stored image files.

#### Barnes circular maze test

The Barnes circular maze test was conducted on "dry land", a white circular surface, 1.0 m in diameter, with 12 holes equally spaced around the perimeter (O'Hara & Co., Tokyo, Japan). The circular open field was elevated 75 cm from the floor, and evenly illuminated by overhead fluorescent white room lighting (1000 lux). A black Plexiglas escape box (17 × 13 × 7 cm) containing shredded paper was located under one of the holes. The hole above the escape box represented the target, analogous to the hidden platform in the Morris task. The location of the target was consistent for a given mouse, but was randomized across mice. The maze was rotated daily, with the spatial location of the target unchanged with respect to the distal visual room cues, to prevent a bias based on olfactory or proximal cues within the maze. Three trials per day were conducted for 7 successive days. On day 8, a probe test was conducted without the escape box, to confirm that this spatial task was acquired based on navigation using distal environment room cues. Three trials were conducted immediately after the probe test, and 7 and 36 days later additional probe tests were conducted again. A single retraining was conducted 7 days later after the probe test at the 36-day retention interval. Latency, number of errors, and distance traveled until they located to the target hole, and time spent around each hole were recorded by Image BM software (see 'Image analyses').

#### Image analyses

The applications used for the behavioral studies (Image EP, Image PS, Image OF, and Image BM) were based on the public domain NIH Image program (developed at the U.S. National Institutes of Health, and available on the Internet at  and ImageJ program , which were modified for each test by Tsuyoshi Miyakawa (available through O'Hara & Co., Tokyo, Japan).

### Histological and immunohistochemical procedures

Brain samples fixed with 4% paraformaldehyde were used for immunohistochemistry with anti-α-CaMKII (BD Biosciences, San Jose, CA), anti-p-α-CaMKII (Promega, Madison, WI), and anti-COI (Molecular Probes, Inc., Eugene, OR) antibodies. Frozen sections of the fixed samples were reacted to the primary antibodies, and then visualized with secondary antibodies Alexa Fluor 488 (for anti-α-CaMKII and anti-COI antibodies) and 594 (for anti-p-α-CaMKII) conjugated goat anti-IgGs (Molecular Probes, Inc., Eugene, OR). The sections were stained with DAPI to visualize all nuclei. For Kluver-Barrera (KB) staining and immunohistochemistry with an anti-NMDA receptor 1 antibody, brain tissues fixed in 4% formaldehyde were used. Paraffin sections (10 μm thick) of the fixed samples were stained with KB method. The sections were also reacted to an anti-NMDA receptor 1 antibody (Acris Antibodies GmbH, Hiddenhausen, Germany) followed by a secondary antibody, biotinylated goat anti-IgGs, and VECTASTAIN ABC kit (Vector Laboratories, Inc., Burlingame, CA). The sections stained with anti-NMDA receptor 1 antibody were counterstained with hematoxylin to visualize all nuclei. Quantification of COI-, α-CaMKII-, and p-α-CaMKII-positive signals were performed by ImageJ program .

### Analysis of complex IV (COX) activity

Estimation of COX activity was carried out by examining the rate of cyanide-sensitive oxidation of reduced cytochrome *c *[36] with modifications. Biochemical analysis was based on the procedure described before [37].

### Western blotting

To detect α-CaMKII protein, frozen visual cortex regions were lysed on ice in 2% SDS (Wako, Osaka, Japan), 50 mM Tris-HCl (pH 6.8), 10% Glycerol (Wako, Osaka, Japan), and 5% 2-Mercaptoethanol (Wako, Osaka, Japan). After centrifugation, the supernatant was used as a sample. Proteins were resolved by SDS-PAGE under reducing conditions. The resolved proteins were transferred electrophoretically to a nitrocellulose membrane (GE Healthcare Bio-Sciences KK, Tokyo, Japan). After incubation with phosphate-buffered saline (PBS, pH 7.4) in 1% Bovine serum albumin (BSA; Sigma-Aldrich, St. Louis, MO) for at least 1 h at room temperature, the membrane was incubated with an anti-α-CaMKII antibody (BD Biosciences, San Jose, CA) for 1 h at room temperature, washed extensively with PBS in 0.1% Tween-20 (ICN Biomedicals Inc., Aurora, OH), and then incubated with biotinylated goat anti-IgGs, and VECTASTAIN ABC kit (Vector Laboratories, Inc., Burlingame, CA). Proteins were detected using Immunostaining HRP-1000 (Konica Minolta, Tokyo, Japan). For loading controls, incubated with a monoclonal anti-β-actin antibody (Sigma-Aldrich, St. Louis, MO) followed by incubation with a horseradish peroxidase-conjugated goat anti-mouse IgG. Each measurement was repeated three times, and the expression of α-CaMKII protein was quantified. Quantification of α-CaMKII protein was performed by ImageJ program .

### Real-time monitoring RT-PCR

Total RNA was extracted by ISOGEN (Nippon Gene, Tokyo, Japan) from frozen organs in mouse brain visual cortex. RNA samples were subjected to DNase I treatment (Invitrogen, Carlsbad, CA) to eliminate DNA contaminants, and reverse transcribed using Oligo (dT)_12–18 _Primer (Invitrogen, Carlsbad, CA), 10 mM dNTP Mix (Invitrogen, Carlsbad, CA), 0.1 M DTT (Invitrogen, Carlsbad, CA), RNase Out Recombinant Ribonuclease Inhibiter (Invitrogen, Carlsbad, CA), and SuperScript II RNase H-Reverse Transcriptase (Invitrogen, Carlsbad, CA). cDNA samples were subjected to RNaseH treatment (Invitrogen, Carlsbad, CA). Real-time monitoring PCR was performed with SYBR Green PCR Master Mix and an ABI PRISM 7900HT sequence detection system (Applied Biosystems, Foster City, CA). The primer set specific for α-CaMKII was GTCGGAATTCCATCCTCACCACTATGCTG and AAGGATCCATCGATGAAAGTCCAGGCCC. Quantitatve-PCR for β-actin mRNA was also performed on the same samples, to correct for any residual differences in the initial level of RNA in the specimens. Results were then normalized using β-actin mRNA levels in the same samples. The primer set specific for β-actin was GGTCATCACTATTGGCAACGAG and GTCAGCAATGCCTGGGTACA. Each measurement was repeated three times, and the expression levels of α-CaMKII mRNA were calculated.

### Statistical analyses

Statistical analyses were performed by using a commercially available software package (STATVIEW, SAS Institute, Cary, NC) or the data analyses add-in for Microsoft Excel. One-way ANOVA followed by Student's t-tests were used for Figures 1D, 1E, 2A, 2B, 4C, 4D, 5C, 6A, and 6B. Two-way repeated-measures ANOVA followed by the two-tailed Student's t-tests were used for Figures 1C, 1F, 2C, and 3A. Paired comparisons t-tests were used for Figure 3B. The relationship between latency, number of errors, or distance and proportion of ΔmtDNA in tails followed by calculating the Pearson's correlation coefficient were used for Figure 3C. Values with *P *< 0.05 were considered significant.

## Competing interests

The authors declare that they have no competing interests.

## Authors' contributions

JIH is responsible for the original concept of this study. KN, TM, HY, and JIH designed the study. AS, KN, and HY generated and provided the mito-mouse. DT, KN, KT, AK, and AS performed the behavioral experiments. DT, KN, and EO carried out histological, immunohistochemical, and biochemical experiments. KN, TM, DT, KT, and JIH wrote the manuscript. All authors read and approved the final manuscript.
